# Efficient Removal of Methylene Blue Dye from Aqueous Media Using Facilely Synthesized Magnesium Borate/Magnesium Oxide Nanostructures

**DOI:** 10.3390/molecules29143392

**Published:** 2024-07-19

**Authors:** Asma S. Al-Wasidi, Raed M. Hegazey, Ehab A. Abdelrahman

**Affiliations:** 1 Department of Chemistry, College of Science, Princess Nourah bint Abdulrahman University, Riyadh 11671, Saudi Arabia; 2 Egyptian Petroleum Research Institute, Ahmed El Zumer Street, Nasr City, Hai Al-Zehour, Cairo 11727, Egypt; 3 Department of Chemistry, College of Science, Imam Mohammad Ibn Saud Islamic University (IMSIU), Riyadh 11623, Saudi Arabia; 4 Chemistry Department, Faculty of Science, Benha University, Benha 13518, Egypt

**Keywords:** Mg_3_B_2_O_6_/MgO nanostructures, adsorption, methylene blue dye, analytical influences

## Abstract

Methylene blue dye in water sources can pose health risks to humans, potentially causing methemoglobinemia, a condition that impairs the blood’s ability to carry oxygen. Hence, the current study investigates the synthesis of novel magnesium borate/magnesium oxide (Mg_3_B_2_O_6_/MgO) nanostructures and their efficiency in removing methylene blue dye from aqueous media. The nanostructures were synthesized using the Pechini sol–gel method, which involves a reaction between magnesium nitrate hexahydrate and boric acid, with citric acid acting as a chelating agent and ethylene glycol as a crosslinker. This method helps in achieving a homogeneous mixture, which, upon calcination at 600 and 800 °C, yields Mg_3_B_2_O_6_/MgO novel nanostructures referred to as MB600 and MB800, respectively. The characterization of these nanostructures involved techniques like X-ray diffraction (XRD), Fourier-transform infrared (FTIR) spectroscopy, N_2_ gas analyzer, and field-emission scanning electron microscope (FE-SEM). These analyses confirmed the formation of orthorhombic Mg_3_B_2_O_6_ and cubic MgO phases with distinct features, influenced by the calcination temperature. The mean crystal size of the MB600 and MB800 samples was 64.57 and 79.20 nm, respectively. In addition, the BET surface area of the MB600 and MB800 samples was 74.63 and 64.82 m^2^/g, respectively. The results indicated that the MB600 sample, with its higher surface area, generally demonstrated better methylene blue dye removal performance (505.05 mg/g) than the MB800 sample (483.09 mg/g). The adsorption process followed the pseudo-second-order model, indicating dependency on available adsorption sites. Also, the adsorption process matched well with the Langmuir isotherm, confirming a homogeneous adsorbent surface. The thermodynamic parameters revealed that the adsorption process was physical, exothermic, and spontaneous. The MB600 and MB800 nanostructures could be effectively regenerated using 6 M HCl and reused across multiple cycles. These findings underscore the potential of these nanostructures as cost-effective and sustainable adsorbents for methylene blue dye removal.

## 1. Introduction

Water pollution with organic dyes is predominantly caused by industrial discharges, specifically from sectors such as textiles, leather, paper, and plastics [1,2,3]. These industries frequently use synthetic dyes to color their products and often discharge untreated or inadequately treated effluents into water bodies. This contamination is exacerbated by insufficient regulatory frameworks, inadequate waste management systems, and a lack of awareness about the environmental impacts of dye effluents. Organic dyes impose significant risks to both ecological and human health. Environmentally, these dyes can severely affect aquatic life by reducing light penetration and oxygen levels in water bodies, thus disturbing aquatic ecosystems [4,5]. From a human health perspective, many organic dyes contain carcinogenic, mutagenic, or toxic compounds. For example, exposure to certain azo dyes has been linked to bladder cancer and other health issues [6,7]. Methylene blue, a commonly used organic dye in various industries, is particularly dangerous to human health. Although used medically for certain treatments, its presence in water at high concentrations can cause significant health issues such as increased heart rate, vomiting, diarrhea, and methemoglobinemia—a condition in which oxygen delivery to tissues is impaired [8,9,10]. The removal of organic dyes from water can be achieved through various methods, such as adsorption [11,12], electrochemical processes [13], photocatalytic degradation [14], and biological processes [15]. Adsorption is usually preferred for dye disposal because of its simplicity, cost effectiveness, and high efficiency, especially at low dye concentrations. Unlike other methods, adsorption does not result in secondary pollution and can be effective across a wide range of pH and temperature conditions. It also allows for the possibility of regenerating the adsorbent, further enhancing its economic viability. The adsorption method is particularly important over other dye removal techniques due to its low energy requirements, making it a more sustainable and cost-effective option for treating water contaminated with organic dyes [16,17,18]. Nanometal oxides are particularly effective in the uptake of organic dyes because of their high surface area and unique chemical properties. These materials can interact strongly with dye molecules, leading to higher removal efficiencies [19,20,21,22]. Chen et al. investigated the synthesis and application of a novel Bi_2_MoO_6_ hierarchical microsphere adsorbent. The adsorbent was highly effective in removing organic pollutants such as methylene blue and Congo red from aqueous solutions [23]. Also, Chen et al. developed a highly efficient and recyclable Bi_2_O_2_CO_3_ (BOC) adsorbent modified with surfactants such as hexadecyl trimethyl ammonium bromide (CTAB) and sodium dodecyl sulfate (SDS), achieving superior performance in removing methyl orange dye and Cr(VI) ions. The BOC-CTAB variant demonstrated the highest adsorption capacity, with simple regeneration via photodegradation [24]. The Pechini sol–gel process is crucial for preparing high-quality metal nanooxides. This method allows for precise control over the chemical composition and structure of the resulting nanomaterials, leading to optimized properties for specific applications like dye adsorption. The method involves using a chelating agent like citric acid to bind metal ions and a crosslinker like ethylene glycol to play a crucial role in enhancing the network structure, facilitating the formation of uniform and highly pure metal oxides [21,22]. The current research introduces an innovative aspect through the synthesis of novel Mg_3_B_2_O_6_/MgO nanostructures using the Pechini sol–gel technique. The use of citric acid as a chelating reagent and ethylene glycol as a crosslinker helps in forming nanostructures that are more effective in adsorbing dyes due to their increased surface area. The application of these novel nanostructures to remove methylene blue dye from aqueous solutions represents a significant innovation. These structures have shown superior performance in removing methylene blue from aqueous solutions, demonstrating high adsorption capacities and the potential for reuse after regeneration, highlighting their practical applicability in environmental cleanup efforts. This research not only contributes to the field of water purification but also advances our understanding of material science in creating efficient and sustainable solutions to pressing environmental problems.

## 2. Results and Discussion

### 2.1. Synthesis and Characterization of Mg₃B₂O₆/MgO Nanostructures

The Pechini sol–gel technique for synthesizing Mg_3_B_2_O_6_/MgO nanostructures involves a series of chemical reactions and process steps using citric acid as a chelating reagent, ethylene glycol as a crosslinker, magnesium nitrate hexahydrate as a magnesium source, and boric acid as a boron source. Magnesium nitrate hexahydrate and boric acid are dissolved in water. The magnesium nitrate dissociates to provide Mg^2^⁺ ions, and boric acid reacts with water to form the tetrahydroxyborate ion, B(OH)_4_⁻, as shown in Equations (1) and (2):

Mg(NO_3_)_2_·6H_2_O→Mg^2+^ + 2NO_3_^−^ + 6H_2_O (1)

H_3_BO_3_ + H_2_O→B(OH)_4_^−^ + H^+^ (2)

Citric acid is added to the aqueous solution containing Mg^2^⁺ and B(OH)_4_⁻. Citric acid chelates both magnesium and boron ions, forming a complex. This complexation improves the homogeneity of the metal ion distribution and enhances the stability of the precursor solution, as shown in Equation (3):

Mg^2+^ + B(OH)_4_^−^ + C_6_H_8_O_7_→[MgB(Citrate)] complex + 4H_2_O (3)

Ethylene glycol is then added to the solution to act as a crosslinking agent [25]. It reacts with the carboxyl groups of the citrate in the metal complex, leading to the formation of ester linkages. This polymerization forms a dense and stable gel, which traps the metal ions uniformly throughout the matrix. The viscous gel that forms is then dried. This step removes water and begins to solidify the complex, preparing it for heat treatment. Upon calcination at high temperatures, the organic components of the gel (citrate and ethylene glycol-based polymers) decompose, and the metal ions react to form the desired mixed oxides. The high temperature facilitates the decomposition of the organic matrix and the formation of magnesium borate (Mg_3_B_2_O_6_) and magnesium oxide (MgO), as shown in Equation (4).

Polymeric Network + heat→Mg_3_B_2_O_6_ + MgO + CO_2_ + H_2_O (4)

**Figure 1:**
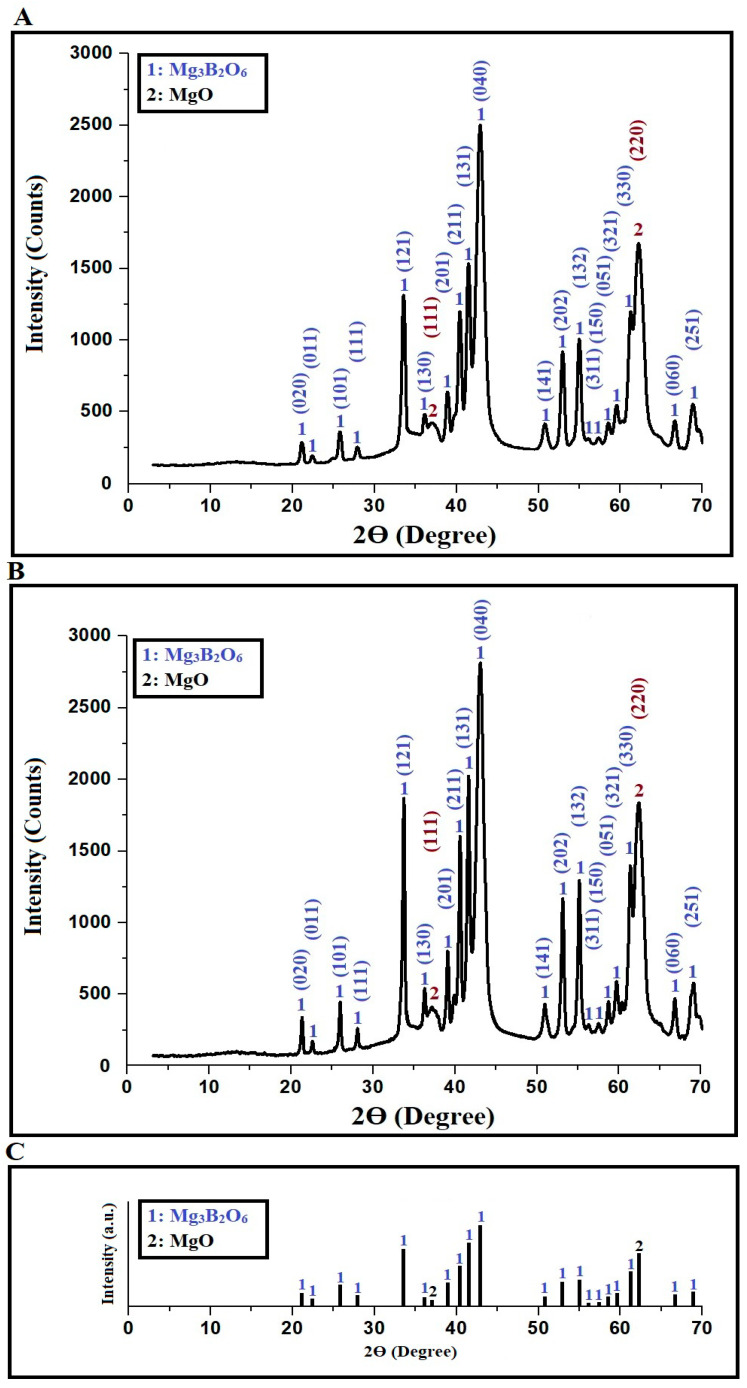
XRD analysis of the MB600 (**A**) and MB800 (**B**) nanostructures. Standard XRD patterns of Mg_3_B_2_O_6_ and MgO phases (**C**).

In the conducted study, the XRD characterization of nanostructures synthesized by the Pechini sol–gel process at 600 and 800 °C was examined, as clarified in Figure 1A,B, respectively. The samples labeled MB600 and MB800 were primarily composed of Mg_3_B_2_O_6_ (orthorhombic system, JCPDS No. 00-038-1475) and MgO (cubic system, JCPDS No. 01-086-8571), as clarified in Figure 1C. It was observed that a rise in the synthesis temperature from 600 °C to 800 °C led to a notable increment in the average crystal size from 64.57 nm to 79.20 nm. These average crystal sizes were calculated by the Scherrer formula [26]. This suggests that the thermal conditions under which the composites were synthesized played a significant role in influencing the crystal growth processes. The higher temperature likely facilitated more energy for the atoms to migrate and arrange into a more stable crystalline structure, resulting in larger crystal sizes. The 2Ɵ° (hkl) values of 21.11° (020), 22.39° (011), 25.71° (101), 27.91° (111), 33.52° (121), 36.09° (130), 38.93° (201), 40.39° (211), 41.39° (131), 42.87° (040), 50.77° (141), 52.88° (202), 54.99° (132), 56.09° (311), 57.29° (150), 58.57° (051), 59.49° (321), 61.24° (330), 66.65° (060), and 68.85° (251) correspond to the Mg_3_B_2_O_6_ orthorhombic phase. Similarly, for the MgO phase, the cubic structure was confirmed by the appearance of 2Ɵ° (hkl) values at 36.99° (111) and 62.15° (220). 

**Figure 2:**
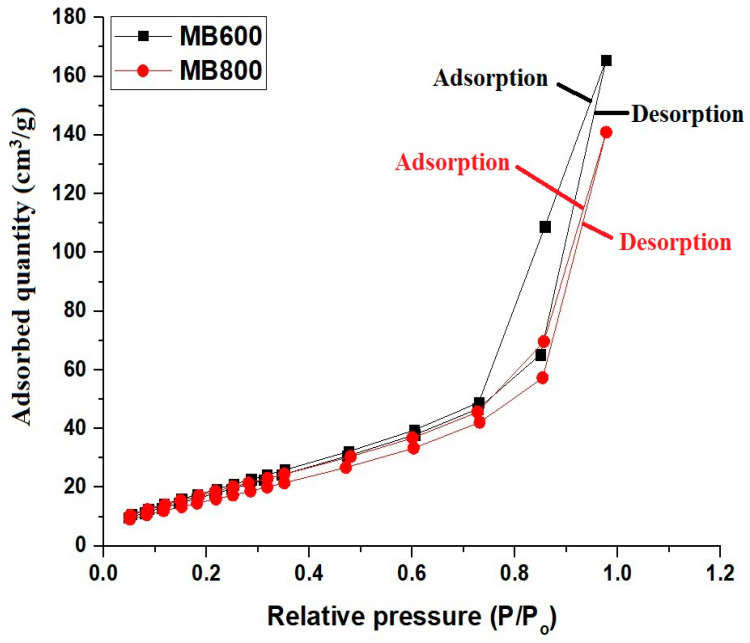
N_2_ adsorption/desorption isotherms of the MB600 and MB800 nanostructures.

Figure 2 describes the N_2_ adsorption/desorption isotherms of the MB600 and MB800 samples, respectively. Analysis of the data reveals that both MB600 and MB800 surface textures exhibited type IV isotherms with distinct hysteresis loops, indicative of mesoporous structures. The BET surface area and total pore volume, as reflected in Table 1, were slightly reduced from MB600 to MB800, with respective values of 74.63 m^2^/g to 64.82 m^2^/g and 0.2568 cm^3^/g to 0.2186 cm^3^/g. This suggests that the thermal treatment corresponding to MB800 may lead to a minor collapse or blockage of pores, thereby reducing the accessible surface area and pore volume [27]. Notably, the mean pore size remained relatively unchanged, with a negligible decrease from 6.88 nm for MB600 to 6.75 nm for MB800. This minor reduction might be attributed to the aforementioned structural alterations.

**Figure 3:**
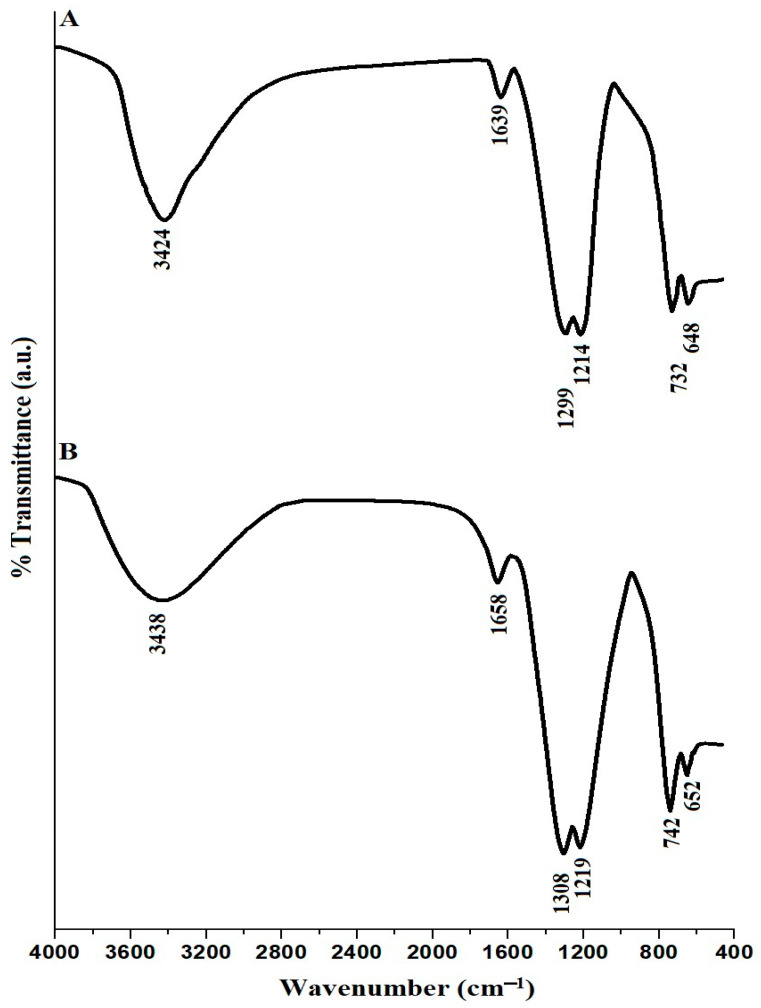
FTIR spectra of the MB600 (**A**) and MB800 (**B**) nanostructures.

Figure 3A,B illustrates the FTIR spectra of the MB600 and MB800 samples, respectively. The stretching vibrations of the Mg-O bond were observed at 648 cm^−1^ for MB600 and 652 cm^−1^ for MB800. Also, the bending vibrations of the B-O-B linkage were detected at 732 cm^−1^ for MB600 and 742 cm^−1^ for MB800. The symmetric stretching vibrations of B-O-B were detected at 1214 cm^−1^ for MB600 and 1219 cm^−1^ for MB800. Moreover, the asymmetric stretching vibrations of B-O-B were detected at 1299 cm^−1^ for MB600 and 1308 cm^−1^ for MB800. The bending vibrations of adsorbed H_2_O were detected at 1639 cm^−1^ for MB600 and 1658 cm^−1^ for MB800. Furthermore, the stretching vibrations of adsorbed H_2_O were detected at 3424 cm^−1^ for MB600 and 3438 cm^−1^ for MB800 [28,29].

**Figure 4:**
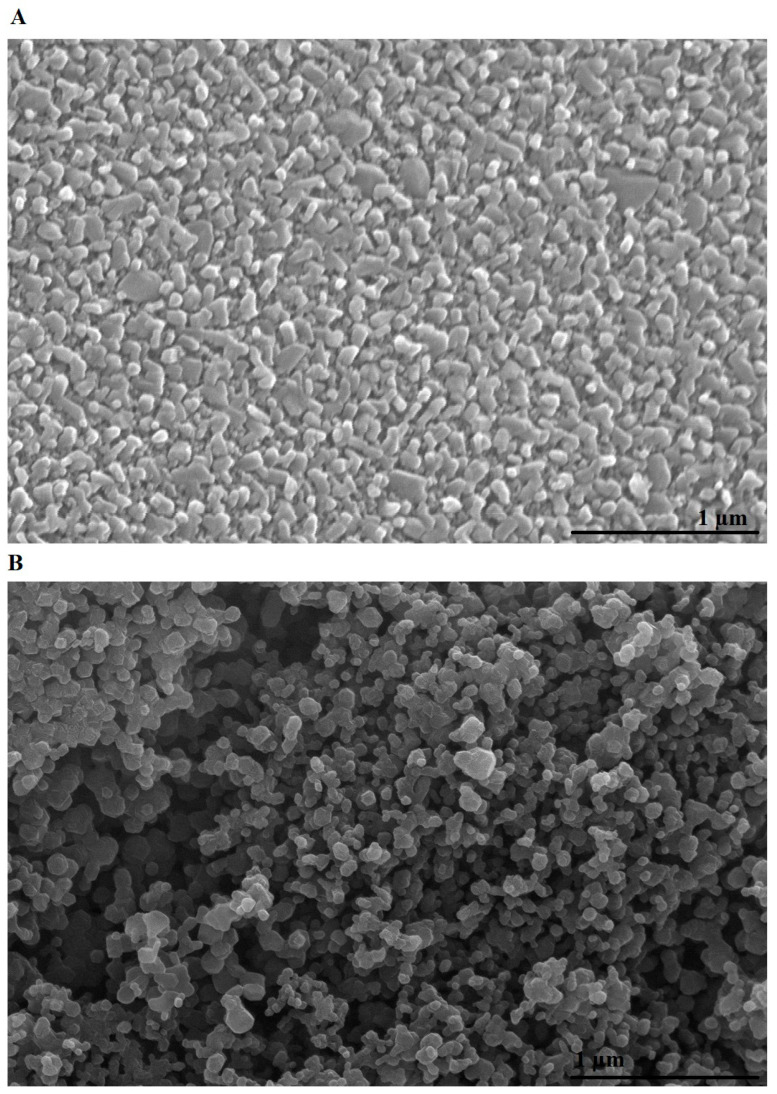
FE-SEM images of the MB600 (**A**) and MB800 (**B**) nanostructures.

Figure 4 presents the field-emission scanning electron microscopy (FE-SEM) images of the MB600 (Figure 4A) and MB800 (Figure 4B) samples, providing insight into the morphological distinctions influenced by the varying treatment temperatures. In Figure 4A, a uniform distribution of fine, densely packed particles with an average diameter of 78.54 nm is observed, indicative of the lower thermal influence during synthesis. On the contrary, Figure 4B reveals the impact of higher thermal treatment, where the particles exhibit coalescence, resulting in larger, more distinct clusters with increased interparticle spaces and an average diameter of 90.01 nm.

### 2.2. Removal of Methylene Blue Dye from Aqueous Solutions

#### 2.2.1. Influence of pH

**Figure 5:**
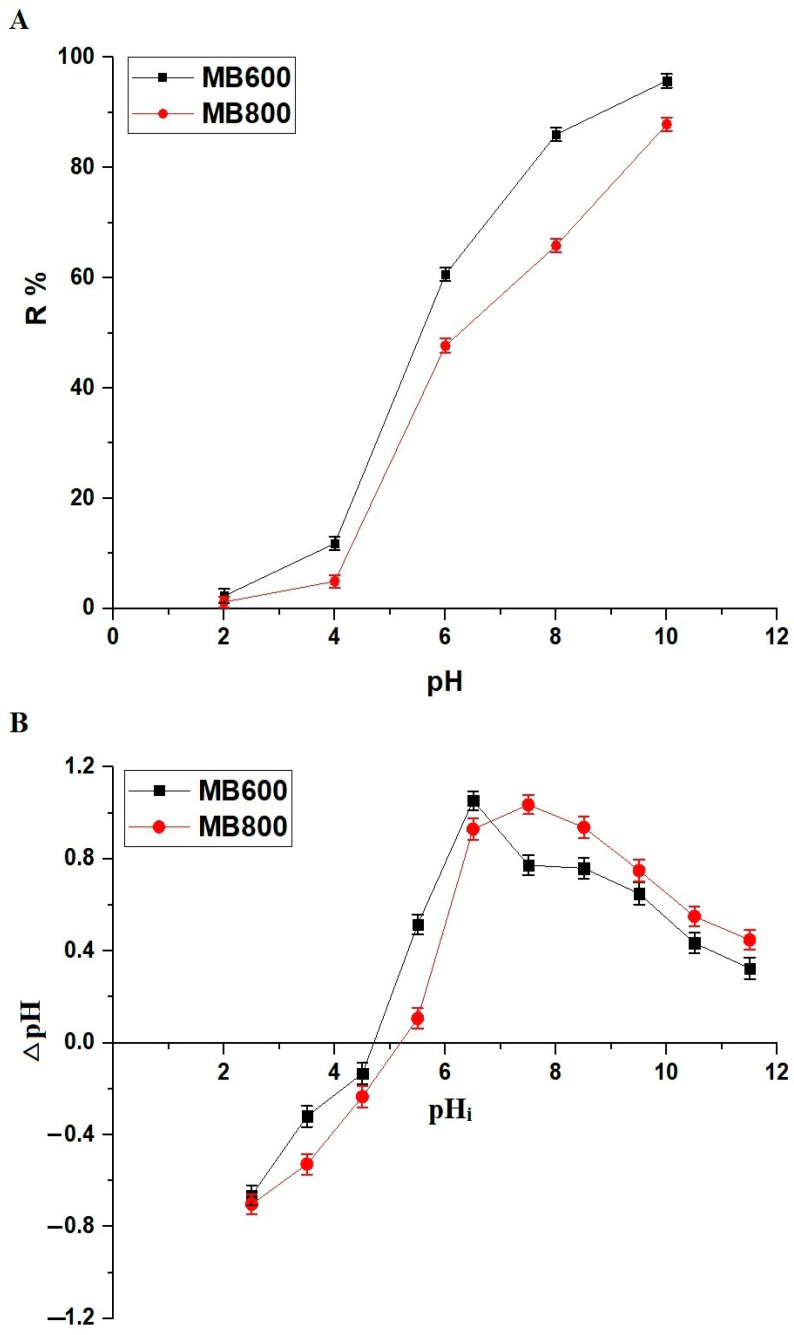
The relationship between pH and the percentage removal of methylene blue dye by the MB600 and MB800 adsorbents (**A**). Experimental conditions: contact time = 180 min, initial dye concentration = 250 mg/L, volume of dye = 100 mL, pH range = 2–10, adsorbent dose = 0.05 g, temperature = 298 K. The point of zero charge of the MB600 and MB800 nanostructures (**B**).

**Scheme 1:**
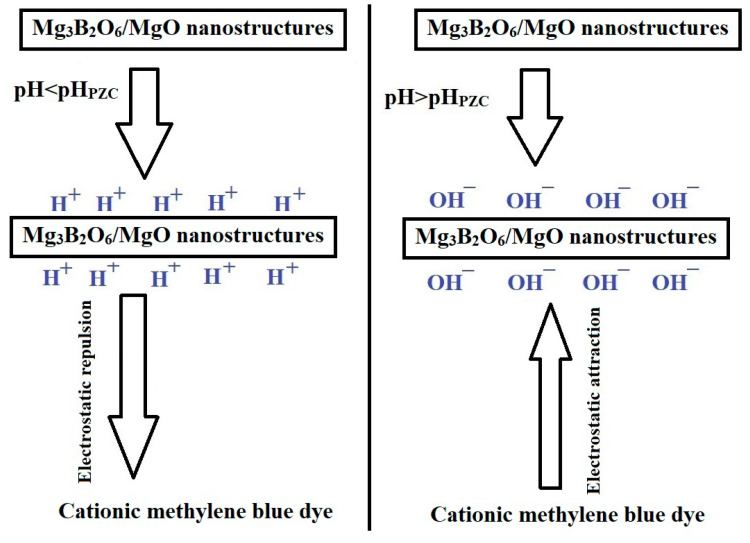
Influence of pH on the surface charge of Mg_3_B_2_O_6_/MgO nanostructures and their interaction with cationic methylene blue dye.

In the presented research, the removal efficiency of methylene blue dye was examined under various pH conditions using the MB600 and MB800 adsorbents, as shown in Figure 5A. It was observed that the percentage removal of the methylene blue dye increased with rising pH values for both adsorbents. At pH 10, the elimination percentage of methylene blue dye utilizing the MB600 and MB800 adsorbents was 95.74 and 87.86%, respectively. The superior performance of the MB600 sample in removing methylene blue dye, compared to the MB800 sample, can be attributed to its larger surface area, which provides an increased number of active sites for dye adsorption. The point of zero charge (pH_PZC_) was estimated to be 4.69 for the MB600 adsorbent and 5.18 for the MB800 adsorbent, as shown in Figure 5B. These values are indicative of the pH at which the adsorbent surface carries no net charge, influencing the adsorption percentage. Above the pH_PZC_, the elimination of methylene blue dye increased because the surface of the adsorbents acquired a net negative charge, enhancing the electrostatic attraction between the adsorbents and the cationic methylene blue dye molecules, as shown in Scheme 1 [30]. Furthermore, below the pH_PZC_, the elimination of methylene blue dye decreased because the surface of the adsorbents acquired a net positive charge, enhancing the electrostatic repulsion between the adsorbents and the cationic methylene blue dye molecules, as shown in Scheme 1 [30].

#### 2.2.2. Influence of Contact Time

**Figure 6:**
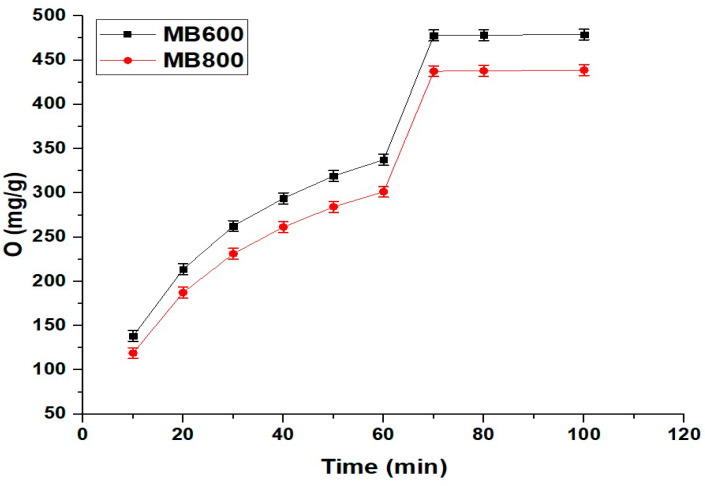
The relationship between time and the adsorption capacity of MB600 and MB800 adsorbents. Experimental conditions: contact time range = 10–100 min, initial dye concentration = 250 mg/L, volume of dye = 100 mL, pH = 10, adsorbent dose = 0.05 g, temperature = 298 K.

In the presented research, the adsorption capacity of the MB600 and MB800 adsorbents toward methylene blue dye was examined at different times, as shown in Figure 6. A gradual increase in the adsorption capacity was noted for both adsorbents as the time progressed from 10 to 70 min. At 70 min, the adsorption capacity of the MB600 and MB800 adsorbents was 477.90 and 437.52 mg/g, respectively. Beyond 70 min, the saturation of active sites prevented any significant increases in the adsorption capacity of the adsorbents.

Pseudo-first-order and pseudo-second-order kinetic models were used to examine the results, as shown in Equations (5) and (6) [30]:

(5) $$\mathrm{Pseudo-first-order}:\log\left(O_{e}-O_{t}\right)=logO_{e}-\frac{F_{1}}{2.303}t$$

(6) $$\mathrm{Pseudo-second-order}:\frac{t}{O_{t}}=\frac{1}{F_{2}O_{e}^{2}}+\frac{1}{O_{e}}t$$

O_e_ and O_t_ represent the quantity of methylene blue dye removed at equilibrium and contact time t, respectively, in the unit of mg/g. Moreover, F_2_ and F_1_ signify the rate constants of the pseudo-second-order and pseudo-first-order models, with the units of g/mg·min and 1/min, respectively. 

**Figure 7:**
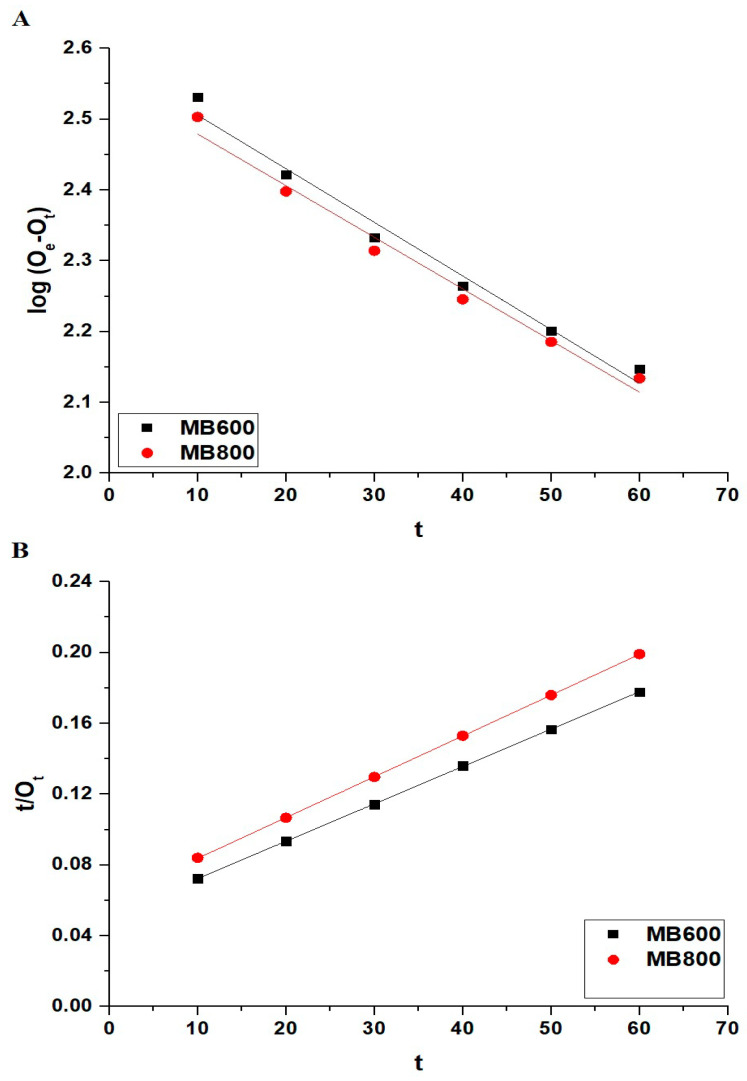
The pseudo-first-order (**A**) and pseudo-second-order (**B**) kinetic models for the disposal of methylene blue dye by the MB600 and MB800 adsorbents.

Figure 7A,B represents the pseudo-first-order and pseudo-second-order models for the elimination of methylene blue dye by the MB600 and MB800 adsorbents, respectively. Also, in Table 2, the kinetic parameters for the elimination of methylene blue dye by MB600 and MB800 adsorbents are presented. Analysis of the kinetic data revealed that the pseudo-second-order model provided a better fit than the pseudo-first-order model, as evidenced by higher R^2^ values of 0.9999 for both adsorbents in the pseudo-second-order model compared to 0.9783 and 0.9787 in the pseudo-first-order model for the MB600 and MB800 adsorbents, respectively. Moreover, the closeness of O_e_ values to the O_EXP_ in the pseudo-second-order model strongly suggests that the adsorption of methylene blue dye onto the MB600 and MB800 adsorbents follows the pseudo-second-order model.

#### 2.2.3. Influence of Temperature

**Figure 8:**
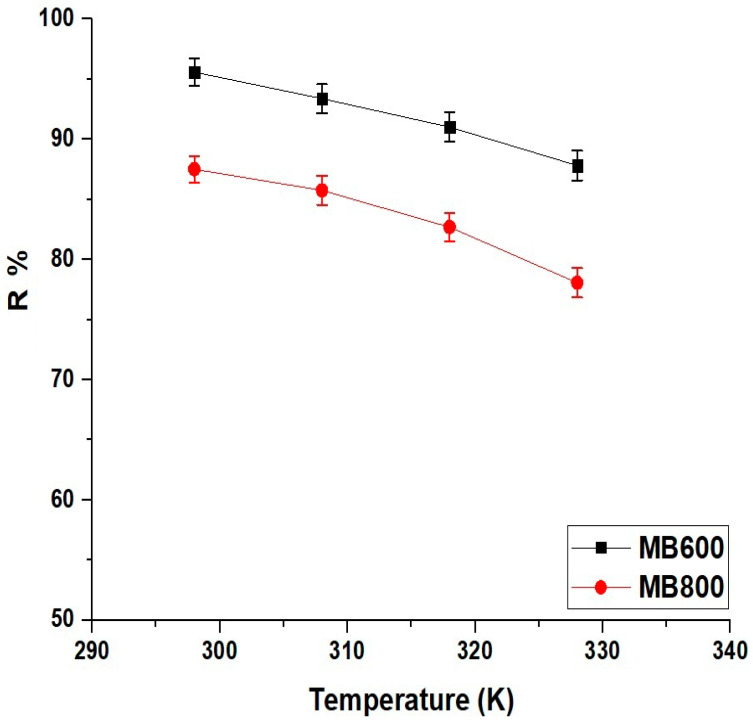
The relationship between time and the percentage removal of methylene blue dye by the MB600 and MB800 adsorbents. Experimental conditions: contact time = 70 min, initial dye concentration = 250 mg/L, volume of dye = 100 mL, pH = 10, adsorbent dose = 0.05 g, temperature range = 298–328 K.

In the presented research, the elimination efficiency of methylene blue dye was examined at different temperatures using the MB600 and MB800 adsorbents, as shown in Figure 8. A gradual decrease in the percentage removal of methylene blue dye was noted for both adsorbents as the temperature progressed from 298 to 328 K.

Equations (7)–(9) were utilized to compute the Gibbs free energy (ΔG°), entropy change (ΔS°), and enthalpy change (ΔH°) for the removal of methylene blue dye by the MB600 and MB800 adsorbents [30].

(7) $$F_{d}=\frac{O_{e}}{C_{e}}$$

(8) $$lnF_{d}=\frac{{△S}^{o}}{R}-\frac{{△H}^{o}}{RT}$$

(9) $${△G}^{o}={△H}^{o}-T{△S}^{o}$$

R, T, and F_d_ are defined as the gas constant, temperature, and distribution coefficient, with the units of kJ/molK, K, and L/g, respectively. 

**Figure 9:**
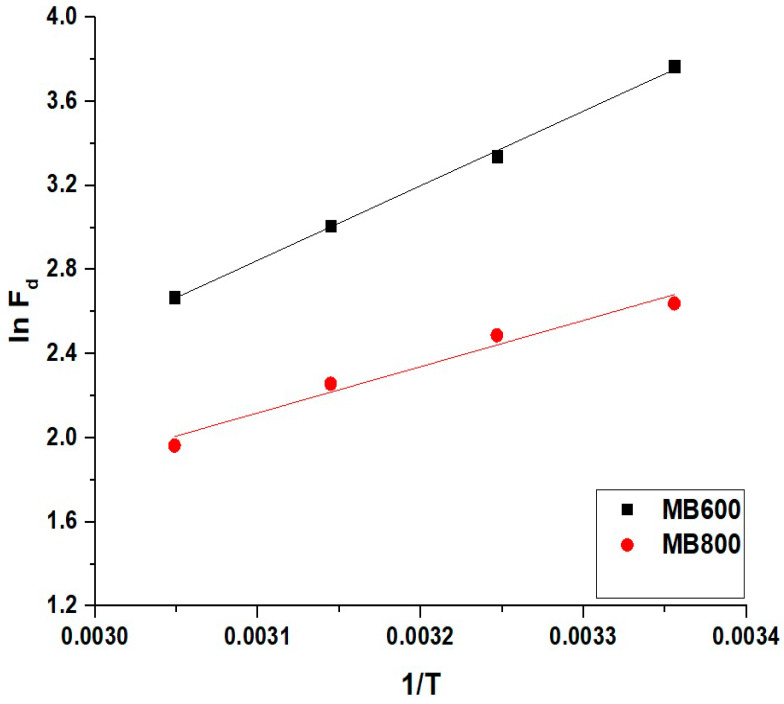
The plot of lnF_d_ against 1/T.

Figure 9 represents the plot of ln F_d_ versus 1/T for the elimination of methylene blue dye using the MB600 and MB800 adsorbents, respectively. Also, in Table 3, the thermodynamic parameters for the removal of methylene blue dye by MB600 and MB800 adsorbents are presented. The positive entropy change (ΔS > 0) indicates that the elimination of methylene blue dye by the MB600 or MB800 adsorbent is feasible, characterized by an increase in randomness at the solid–liquid interface. The enthalpy change (ΔH) is less than 40 kJ/mol, indicating that the adsorption mechanism of methylene blue dye is primarily physical in nature [27]. The negative enthalpy change (ΔH < 0) confirms that the elimination process of methylene blue dye is exothermic. A positive Gibbs free energy change (ΔG > 0) suggests that the adsorption of methylene blue dye occurs spontaneously.

#### 2.2.4. Influence of Adsorbent Dose

**Figure 10:**
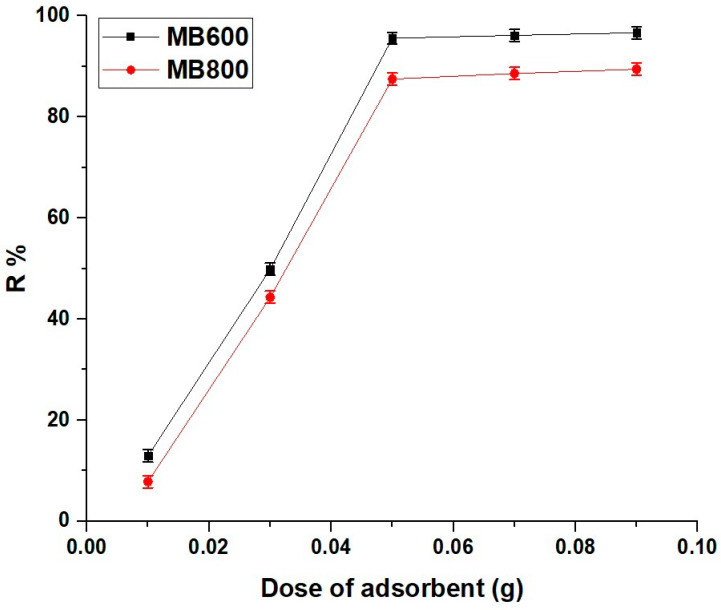
The relationship between adsorbent dose and the percentage removal of methylene blue dye by the MB600 and MB800 adsorbents. Experimental conditions: contact time = 70 min, initial dye concentration = 250 mg/L, volume of dye = 100 mL, pH = 10, adsorbent dose range = 0.01–0.09 g, temperature = 298 K.

For both MB600 and MB800 adsorbents, the percentage removal of methylene blue dye improved significantly as the adsorbent dose increased from 0.01 g to 0.05 g, reaching a plateau beyond this dosage, as shown in Figure 10. This behavior can be attributed to the increase in available adsorption sites with higher adsorbent doses (0.01–0.05 g), leading to enhanced dye uptake. Further additions, beyond 0.05 g, resulted in the aggregation of the adsorbent particles. Hence, there is an approximate stability in the dye removal percentage. Consequently, 0.05 g was selected as the optimal amount for further experiments to maintain a balance between efficiency and practicality. 

#### 2.2.5. Influence of Concentration

**Figure 11:**
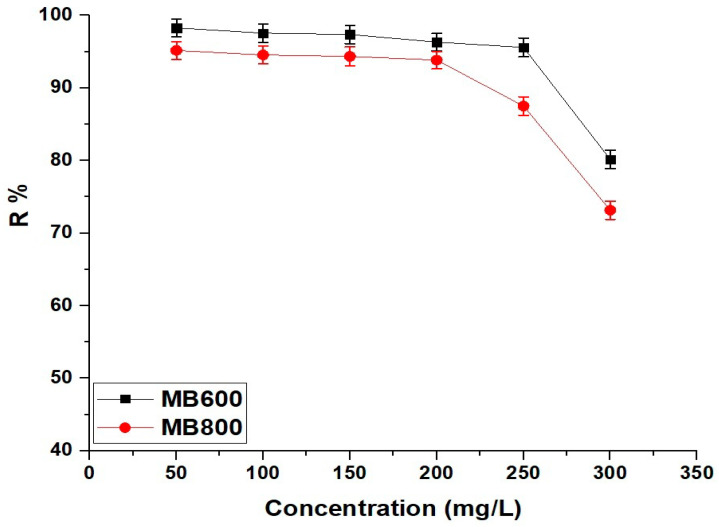
The relationship between initial dye concentration and the percentage elimination of methylene blue dye by the MB600 and MB800 adsorbents. Experimental conditions: contact time = 70 min, initial dye concentration range = 50–300 mg/L, volume of dye = 100 mL, pH = 10, adsorbent dose = 0.05 g, temperature = 298 K.

In the presented research, the elimination efficiency of methylene blue dye was examined at different concentrations using the MB600 and MB800 adsorbents, as shown in Figure 11. A gradual decrease in the percentage removal of methylene blue dye was noted for both adsorbents as the concentration progressed from 50 to 300 mg/L.

The Langmuir and Freundlich equilibrium isotherms were used to examine the results, as shown in Equations (10) and (11) [30]:

(10) $$Freundlich\;isotherm:ln\left(O_{e}\right)=ln\left(F_{3}\right)+\frac{1}{Y}ln(C_{e})$$

(11) $$Langmuir\;isotherm:\frac{C_{e}}{O_{e}}=\frac{1}{F_{4}O_{max}}+\frac{C_{e}}{O_{max}}$$

F_3_ and F_4_ reveal the rate constants of the Freundlich and Langmuir isotherms, with the units of (mg/g) (L/mg)^1/n^) and (L/mg), respectively. The adsorption intensity is represented by 1/Y, and the maximum uptake capacity is denoted as O_max_ (mg/g). The Freundlich equilibrium isotherm was used to calculate O_max_, as outlined in Equation (12) [30].

(12) $$O_{max}=F_{3}\left(C_{o}^{1/Y}\right)$$

**Figure 12:**
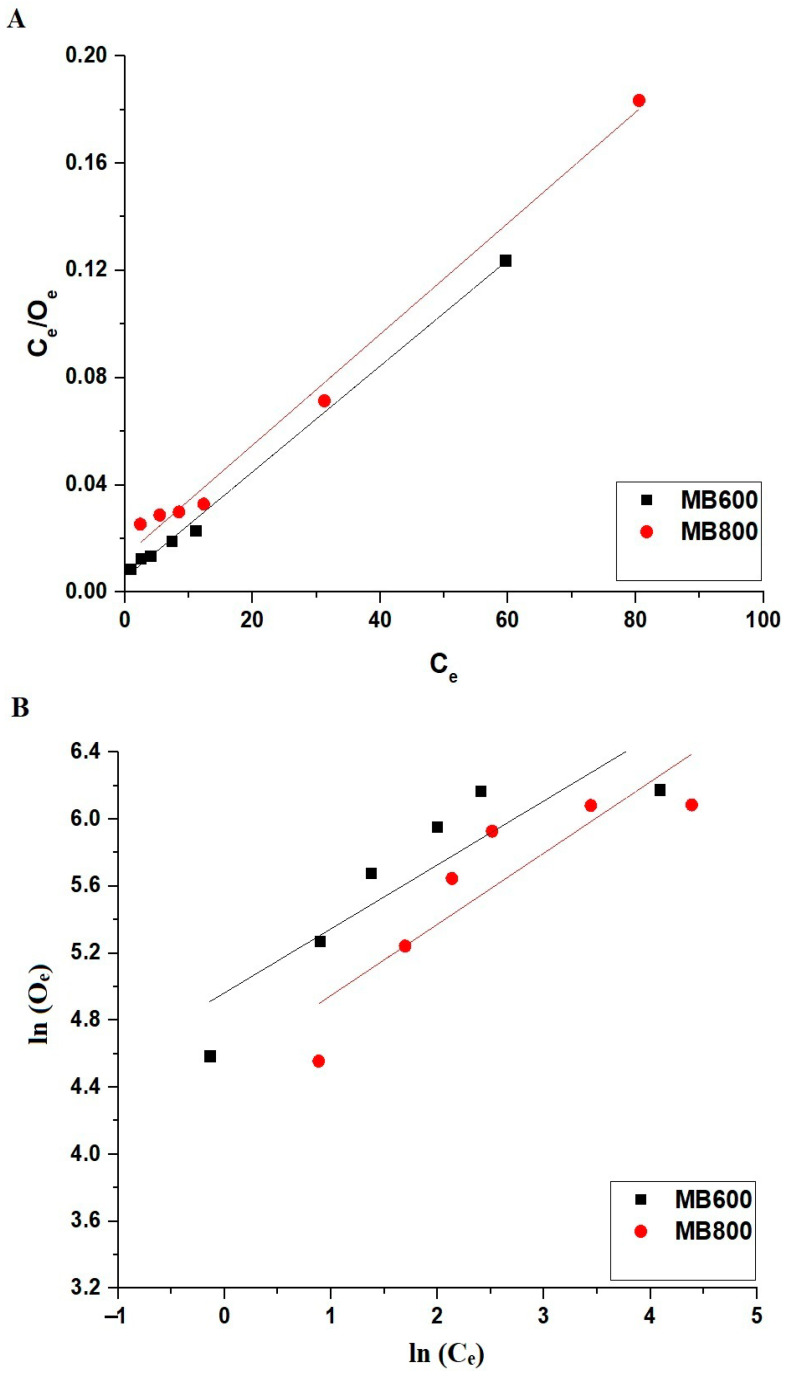
The Langmuir (**A**) and Freundlich (**B**) equilibrium isotherms for the elimination of methylene blue dye by the MB600 and MB800 adsorbents.

Figure 12A,B represents the Langmuir and Freundlich isotherms for the elimination of methylene blue dye by the MB600 and MB800 adsorbents, respectively. Also, in Table 4, the equilibrium parameters for the removal of methylene blue dye by MB600 and MB800 adsorbents are presented. Analysis of the equilibrium data revealed that the Langmuir isotherm provided a better fit than the Freundlich isotherm, as evidenced by higher R^2^ values of 0.9966 and 0.9896 for both adsorbents in the Langmuir isotherm compared to 0.7398 and 0.7362 in the Freundlich isotherm for the MB600 and MB800 adsorbents, respectively. 

In the presented study, the adsorption capacities of various adsorbents toward methylene blue dye were compared, as summarized in Table 5 [31,32,33,34,35,36,37,38]. Among the evaluated adsorbents, the highest adsorption capacity was observed for MB600, with a value of 505.05 mg/g, closely followed by MB800, which exhibited a capacity of 483.09 mg/g. These values were significantly higher when compared to other adsorbents. The superior performance of the newly studied MB600 and MB800 adsorbents suggests their potential for enhanced dye removal in wastewater treatment applications. These findings highlight the ongoing advancements in adsorbent technology and the critical role of material engineering in environmental remediation efforts. The enhanced adsorption capacities can be attributed to the synergistic interaction between Mg_3_B_2_O_6_ and MgO. Mg_3_B_2_O_6_ provides a high surface area and mesoporosity, facilitating dye molecules’ access to active sites. Meanwhile, MgO contributes to structural stability and provides additional active sites for adsorption. This combination results in a material with superior adsorption characteristics compared to the individual components alone. The interaction between these phases enhances the overall performance by optimizing the available surface area, pore structure, and adsorption sites, leading to higher removal efficiencies.

#### 2.2.6. Effect of Regeneration and Reusability

**Figure 13:**
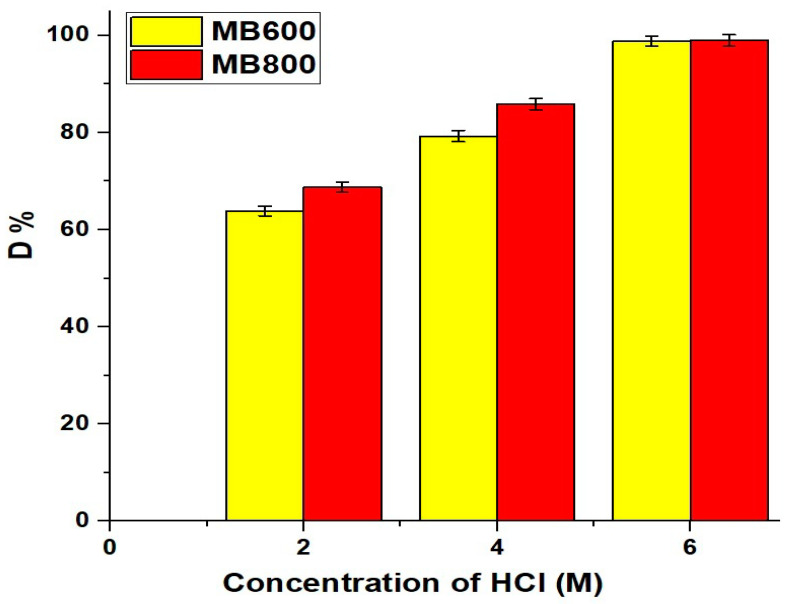
Desorption efficiency of methylene blue dye from the MB600 and MB800 adsorbents at varying concentrations of HCl. Experimental conditions: contact time = 70 min, initial dye concentration range = 250 mg/L, volume of dye = 100 mL, pH = 10, adsorbent dose = 0.05 g, temperature = 298 K, concentration of HCl eluting agent range = 2–6 M, volume of HCl = 50 mL, desorption time = 30 min.

In the current work, the effectiveness of different concentrations of HCl as an eluting agent for the desorption of methylene blue dye from the MB600 and MB800 adsorbents was evaluated, as shown in Figure 13. It was found that, as the concentration of HCl improved, the percentage of dye desorbed also increased for both types of adsorbents. The highest desorption efficiency was achieved at a concentration of 6 M, where the desorption percentages were 98.82% for the MB600 adsorbent and 99.03% for the MB800 adsorbent. These results suggest that the desorption capacity is powerfully dependent on the acidity of the eluting solution. The higher desorption percentages at increased HCl concentrations can be attributed to the enhanced disruption of interactions between the methylene blue dye molecules and the adsorbent surfaces. The near-complete desorption at the highest concentration highlights the potential for regenerating and reusing these adsorbents. This study emphasizes the importance of optimizing eluent concentration for effective dye recovery and adsorbent reuse. The findings indicate that both MB600 and MB800 adsorbents exhibit excellent potential for cyclic usage with effective desorption capabilities.

**Figure 14:**
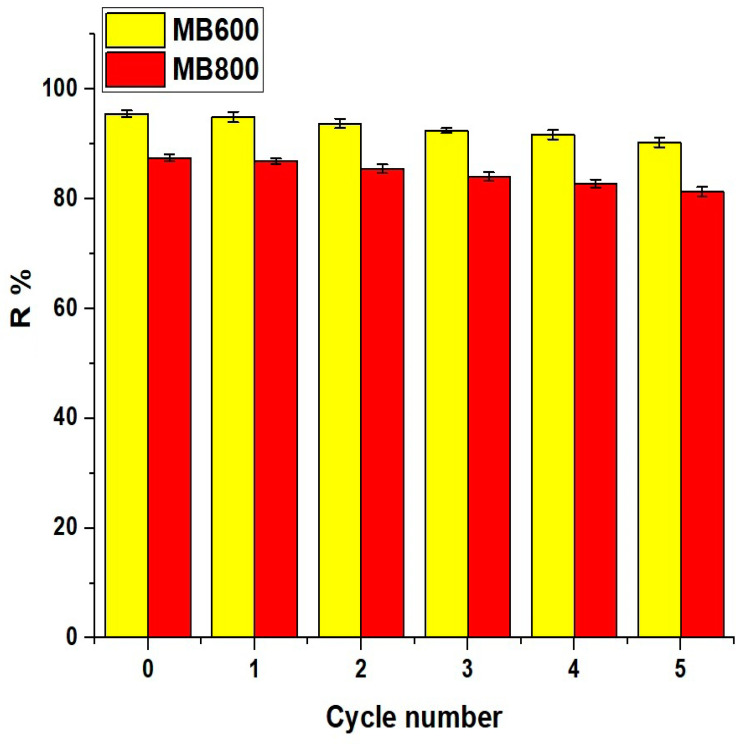
Reusability of the MB600 and MB800 adsorbents over multiple cycles for the adsorption of methylene blue dye. Experimental conditions: quantity adsorbed (477.90 mg/g for MB600 or 437.52 mg/g for MB800), initial dye concentration = 250 mg/L, volume of dye solution = 100 mL, amount of adsorbent = 0.05 g, pH = 10, contact adsorption time = 70 min, adsorption temperature = 298 K, concentration of HCl eluting agent = 6 M, volume of HCl = 50 mL, desorption time = 30 min.

The reusability of the MB600 and MB800 adsorbents for the removal of methylene blue dye was systematically investigated over several cycles, as shown in Figure 14. The removal percentage remained relatively stable over five cycles, indicating that the adsorption efficiency of the adsorbents was not significantly affected by repeated use. The sustained adsorption efficiency over multiple cycles underscores the potential for the cost-effectiveness and long-term applicability of these adsorbents in water treatment.

## 3. Experimental Section

### 3.1. Materials

Boric acid (H_3_BO_3_), magnesium nitrate hexahydrate (Mg(NO_3_)_2_·6H_2_O), ethylene glycol (C_2_H_6_O_2_), citric acid (C_6_H_8_O_7_), sodium hydroxide (NaOH), hydrochloric acid (HCl), methylene blue dye (C_16_H_18_N_3_SCl), and potassium chloride (KCl) were purchased from Sigma-Aldrich Company (St. Louis, MO, USA). Each substance was used as received without further purification.

### 3.2. Synthesis of Mg_3_B_2_O_6_/MgO Nanostructures

**Scheme 2:**
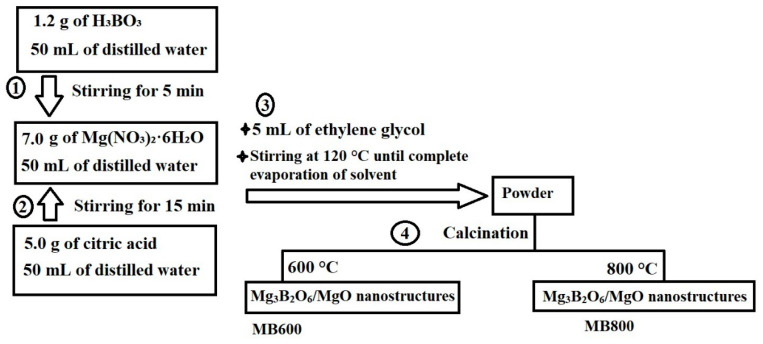
Synthesis method of Mg_3_B_2_O_6_/MgO nanostructures.

Initially, 7.0 g of magnesium nitrate hexahydrate was solubilized in 50 mL of distilled water. Following this, 1.2 g of boric acid was dissolved in another 50 mL of distilled water, and this solution was then added to the magnesium nitrate solution with constant stirring for 5 min. Subsequently, 5.0 g of citric acid was dissolved in 50 mL of distilled water, and this solution was added to the previous mixture with continuous stirring for about 15 min. Afterward, 5 mL of ethylene glycol was incorporated into the previous mixture, which was then stirred at 120 °C until the solvent had completely evaporated. The resultant powder was calcined at temperatures of 600 °C and 800 °C to produce Mg_3_B_2_O_6_/MgO nanostructures, which were referred to as MB600 and MB800, respectively. Scheme 2 presents the synthesis method of Mg_3_B_2_O_6_/MgO nanostructures. 

### 3.3. Instrumentation

The Fourier-transform infrared (FTIR) spectra of the MB600 and MB800 samples were obtained using an FTIR spectrometer (Cary 630, Agilent Technologies, Santa Clara, CA, USA). The surface morphology of the MB600 and MB800 products was examined using a field-emission scanning electron microscope (Quanta 250 FEG, Thermo Fisher Scientific, Waltham, MA, USA). Gas adsorption–desorption analysis was conducted using an N_2_ gas analyzer (NOVAtouch, Quantachrome, Boynton Beac, FL, USA). Before the analysis, the samples were degassed at 120 °C for 12 h to remove any adsorbed gases or moisture. Approximately 0.15 g of the sample was placed into the analysis cell for the measurement. X-ray diffraction (XRD) patterns were obtained using an X-ray diffractometer (X’Pert PRO, PANalytical, Almelo, The Netherlands). The XRD peaks were identified using the X’Pert HighScore software 4.9 and the JCPDS database.

### 3.4. Removal of Methylene Blue Dye from Aqueous Media

The removal efficiency of methylene blue dye was assessed using MB600 or MB800 adsorbents across various conditions, as shown in Table 6. Solutions of 250 mg/L methylene blue dye concentration and 100 mL volume were treated with 0.05 g of adsorbent while varying the pH from 2 to 10, the contact time from 10 to 100 min, and the temperature from 298 to 328 K, as clarified in Table 1. The influence of different dye concentrations extending from 50 to 300 mg/L was also explored, with all experiments maintaining a constant volume and adsorbent amount. To study the effect of the adsorbent dose, 100 mL of 250 mg/L methylene blue dye solution was treated with varying adsorbent dosages from 0.01 to 0.09 g according to the experimental conditions in Table 6. Experimental procedures involved stirring the solutions for specified times, removing the adsorbent by centrifugation, and measuring the residual methylene blue dye concentration at 660 nm using a UV–Vis spectrophotometer (V-670, Jasco, Tokyo, Japan) to determine the adsorption efficiency. Moreover, all experiments were performed in triplicate.

Two primary equation, Equations (13) and (14), were used to quantify the efficiency of the synthesized adsorbents in removing methylene blue dye from aqueous media [39,40].

(13) $$Adsorption\;Capacity\left(O,mg/g\right):O=\left(C_{o}-C_{e}\right)\times \frac{V}{W}$$

(14) $$Removal\;Efficiency\left(R\%\right):R\%=\frac{C_{o}-C_{e}}{C_{o}}\times 100$$

C_o_ serves as the initial concentration of the methylene blue dye in the solution (mg/L). C_e_ serves as the equilibrium concentration of the methylene blue dye in the solution after adsorption (mg/L). V serves as the volume of the methylene blue dye solution (L). W serves as the mass of the adsorbent (g). 

Hydrochloric acid (HCl) in concentrations of 2, 4, and 6 M was utilized as the eluting agent, each at a volume of 50 mL, to regenerate the methylene blue dye-laden adsorbents. 

Equation (15) was used to quantify the desorption efficiency of methylene blue dye from the adsorbent [41].

(15) $$Desorption\;Efficiency\left(D\%\right)=\frac{100C_{d}V_{d}}{(C_{o}-C_{e})V}$$

C_d_ serves as the concentration of the methylene blue dye in the desorption solvent (mg/L). V_d_ serves as the volume of the desorption solvent (L). 

After regeneration, the reusability of the adsorbents was evaluated through five repeated cycles to determine their long-term performance. Each cycle entailed treating 100 mL of a 250 mg/L methylene blue dye solution with 0.05 g of adsorbent. The experimental conditions for each cycle were uniformly maintained, including a contact time of 70 min, a temperature of about 298 K, and a pH of 10.

### 3.5. Point of Zero Charge (pH_PZC_) of the MB600 and MB800 Adsorbents

To estimate the point of zero charge (pH_PZC_) for the MB600 and MB800 nanostructures, the batch method was employed [30]. The beginning pH values (pH_i_) of 50 mL of KCl solutions were adjusted and recorded before adding 0.1 g of the adsorbent. After 12 hrs of stirring, the final pH values (pH_f_) of the solutions were recorded. The difference between the final and initial pH values (∆pH) was plotted against the initial pH values (pH_i_). Hence, the point at which the plot of ∆pH versus pH_i_ crosses the horizontal axis indicates the pH_PZC_. 

## 4. Conclusions

This study explores the synthesis of innovative magnesium borate/magnesium oxide (Mg_3_B_2_O_6_/MgO) nanostructures and evaluates their effectiveness in removing methylene blue dye from aqueous media. The nanostructures were produced using the Pechini sol–gel method, a process involving a reaction between boric acid and magnesium nitrate hexahydrate, with citric acid as a chelating agent and ethylene glycol as a crosslinker. The nanostructures, which were produced at 600 and 800 °C, were labeled as MB600 and MB800, respectively. Also, the average crystal sizes of the MB600 and MB800 samples were 64.57 and 79.20 nm, respectively. In addition, the BET surface areas of the MB600 and MB800 products were 74.63 and 64.82 m^2^/g, respectively. Results showed that the MB600 sample, with a higher surface area, typically outperformed the MB800 in methylene blue dye removal, achieving adsorption capacities of 505.05 and 483.09 mg/g, respectively. Both MB600 and MB800 nanostructures were successfully regenerated using 6 M HCl and showed high reusability over multiple cycles. These results highlight the potential of these nanostructures as economical and sustainable adsorbents for methylene blue dye removal.

## Figures and Tables

**Table 1:** Surface textures of the MB600 and MB800 nanostructures.

Surface Textures	MB600	MB800
BET surface area (m^2^/g)	74.63	64.82
Total pore volume (cm^3^/g)	0.2568	0.2186
Mean pore size (nm)	6.88	6.75

**Table 2:** Kinetic parameters for methylene blue dye elimination by the MB600 and MB800 adsorbents.

Adsorbent	O_Exp_ (mg/g)	Pseudo-First-Order			Pseudo-Second-Order		
		F_1_ (1/min)	O_e_ (mg/g)	R^2^	F_2_ (g/mg·min)	O_e_ (mg/g)	R^2^
MB600	477.90	0.01743	381.38	0.9783	8.6870 × 10^−5^	473.93	0.9999
MB800	437.52	0.01679	356.30	0.9787	8.7107 × 10^−5^	434.78	0.9999

**Table 3:** Thermodynamic parameters for methylene blue dye elimination by the MB600 and MB800 adsorbents.

Adsorbent	ΔS° (kJ/molK)	ΔH° (kJ/mol)	ΔG° (kJ/mol)			
			298	308	318	328
MB600	0.06812	−29.65	−49.95	−50.63	−51.31	−51.99
MB800	0.03926	−18.38	−30.08	−30.47	−30.86	−31.26

**Table 4:** Equilibrium parameters for methylene blue dye disposal by the MB600 and MB800 adsorbents.

Adsorbent	Langmuir			Freundlich			
	O_max_ (mg/g)	F_4_ (L/mg)	R^2^	O_max_ (mg/g)	F_3_ (mg/g) (L/mg)^1/n^	1/Y	R^2^
MB600	505.05	0.3646	0.9966	1177.25	143.21	0.3815	0.7398
MB800	483.09	0.1529	0.9896	961.68	92.15	0.4248	0.7362

**Table 5:** Comparison of maximum uptake capacities for various adsorbents toward methylene blue dye.

Adsorbent	O_max_ (mg/g)	Ref.
γ-Al_2_O_3_	175.40	[31]
Amine-functionalized sodium alginate hydrogel	400.00	[32]
Betaine-modified magnetic nanoparticles	136.00	[33]
Chitosan/graphene oxide composite	81.50	[34]
Hydrolyzed-polyacrylamide-modified diatomite waste	37.12	[35]
Activated carbon	219.90	[36]
Chitosan/epichlorohydrin/zeolite composite	156.10	[37]
Magadiite–chitosan composite beads	45.25	[38]
MB600	505.05	This study
MB800	483.09	This study

**Table 6:** Experimental conditions for removing methylene blue dye using the MB600 and MB800 adsorbents.

Influence	Concentration of Methylene Blue Dye (mg/L)	Volume of Methylene Blue Dye Solution (mL)	Quantity of Adsorbent (g)	Contact Time (min)	Temperature (K)	pH
pH	250	100	0.05	180	298	2–10
Time	250	100	0.05	10–100	298	10
Temperature	250	100	0.05	70	298–328	10
Dose of adsorbent	250	100	0.01–0.09	70	298	10
Concentration of dye	50–300	100	0.05	70	298	10

## Data Availability

Data are contained within the article.
